# PTEN nuclear translocation enhances neuronal injury after hypoxia-ischemia via modulation of the nuclear factor-κB signaling pathway

**DOI:** 10.18632/aging.203141

**Published:** 2021-06-10

**Authors:** Jing Zhao, Linlin Yin, Lin Jiang, Li Hou, Ling He, Chunyan Zhang

**Affiliations:** 1 Department of Neonatology, Affiliated Hospital of North Sichuan Medical College, Nanchong 637000, Sichuan, China

**Keywords:** brain damage, hypoxia-ischemia, oxygen and glucose deprivation, phosphatase and tensin homolog deletion from chromosome 10, ERK1/2

## Abstract

The occurrence of hypoxia-ischemia (HI) in the developing brain is closely associated with neuronal injury and even death. However, the underlying molecular mechanism is not fully understood. This study was designed to investigate phosphatase and tensin homolog (PTEN) nuclear translocation and its possible role in rat cortical neuronal damage following oxygen-glucose deprivation (OGD) *in vitro*. An *in vitro* OGD model was established using primary cortical neurons dissected from newborn Sprague-Dawley rats to mimic HI conditions. The PTEN_K13R_ mutant plasmid, which contains a lysine-to-arginine mutation at the lysine 13 residue, was constructed. The nuclei and cytoplasm of neurons were separated. Neuronal injury following OGD was evidenced by increased lactate dehydrogenase (LDH) release and apoptotic cell counts. In addition, PTEN expression was increased and the phosphorylation of extracellular signal-regulated kinase 1/2 (p-ERK1/2) and activation of nuclear factor kappa B (NF-κB) were decreased following OGD. PTEN_K13R_ transfection prevented PTEN nuclear translocation; attenuated the effect of OGD on nuclear p-ERK1/2 and NF-κB, apoptosis, and LDH release; and increased the expression of several anti-apoptotic proteins. We conclude that PTEN nuclear translocation plays an essential role in neuronal injury following OGD via modulation of the p-ERK1/2 and NF-κB pathways. Prevention of PTEN nuclear translocation might be a candidate strategy for preventing brain injury following HI.

## INTRODUCTION

Hypoxia-ischemia (HI) during fetal and neonatal development can cause damage to neurons, leading to neurological defects and even death [1]. Brain damage induced by loss of oxygen and glucose supply is closely associated with cell death via both necrosis and apoptosis [2]. Although there have been major advances in the understanding of the pathologies of HI-induced brain injury, excitotoxic neuronal cell damage and death are not fully understood. Understanding the pathogenesis of HI-induced brain injury is vital for facilitating the discovery of effective therapies. Thus, it is necessary to fully explore the underlying mechanisms of HI-induced neuronal injuries.

Phosphatase and tensin homolog deleted on chromosome 10 (PTEN) is a candidate tumor suppressor gene, found to be frequently deleted or mutated in a variety of human cancers [3–5]. PTEN possesses both protein phosphatase and lipid phosphatase activity [6] and PTEN nuclear translocation induced by PTEN phosphatase activity has been linked to DNA damage in cancer cells [7]. The tumor suppressive function of PTEN is closely related to its nucleocytoplasmic distribution and interactions with the phosphoinositide 3-kinase (PI3K)/AKT serine/threonine kinase (AKT) signaling pathway [8]. Additionally, PTEN has been reported to localize to both the cytoplasm and nucleus of neurons [9], and an imbalance in its nucleocytoplasmic distribution has been linked to neuronal injury following excitotoxicity or ischemia [10, 11]. Our previous studies showed that the inhibition of PTEN prevents neuronal injury after HI by mediating the activity of glycogen synthase kinase 3β (GSK-3β) and AKT [12, 13].

The mitogen-activated protein kinase (MAPK) pathway is another predominant pathway implicated in neuronal injury [14, 15]. Extracellular signal-regulated kinase 1/2 (ERK1/2) is one of the most well-characterized members of the MAPK family [16]. ERK1/2 isoforms have received substantial attention in neurological research [17–19], as many cell types in the central nervous system, including neurons, glia, and endothelial cells, express phosphorylated ERK (p-ERK) after injury [20]. In addition, it has been shown that elevated ERK phosphorylation may protect neurons against ischemic death in rat and mouse brains [21]. Activated nuclear factor kappa B (NF-κB) plays a critical role in the transcriptional response to hypoxia, and has been shown to be regulated by the ERK pathway [22]. Similarly, it has been reported that nuclear PTEN can inhibit NF-κB activation [23]. However, the roles of PTEN in the regulation of ERK phosphorylation and NF-κB activity and their effects on the underlying mechanisms of neuronal injury remain unclear.

Therefore, this study used an oxygen-glucose deprivation (OGD) model established in the primary cortical neurons of newborn Sprague-Dawley rats to mimic HI *in vitro*. The effects of the nuclear translocation of PTEN and the possible roles of PTEN/ERK/NF-κB signaling in cortical neurons after OGD were studied. We anticipate that this study could provide new perspectives into our understanding of the role of PTEN in neuronal injury and therapy development.

## RESULTS

### OGD increases PTEN nuclear translocation in primary cortical neuronal cultures

**Figure 1:**
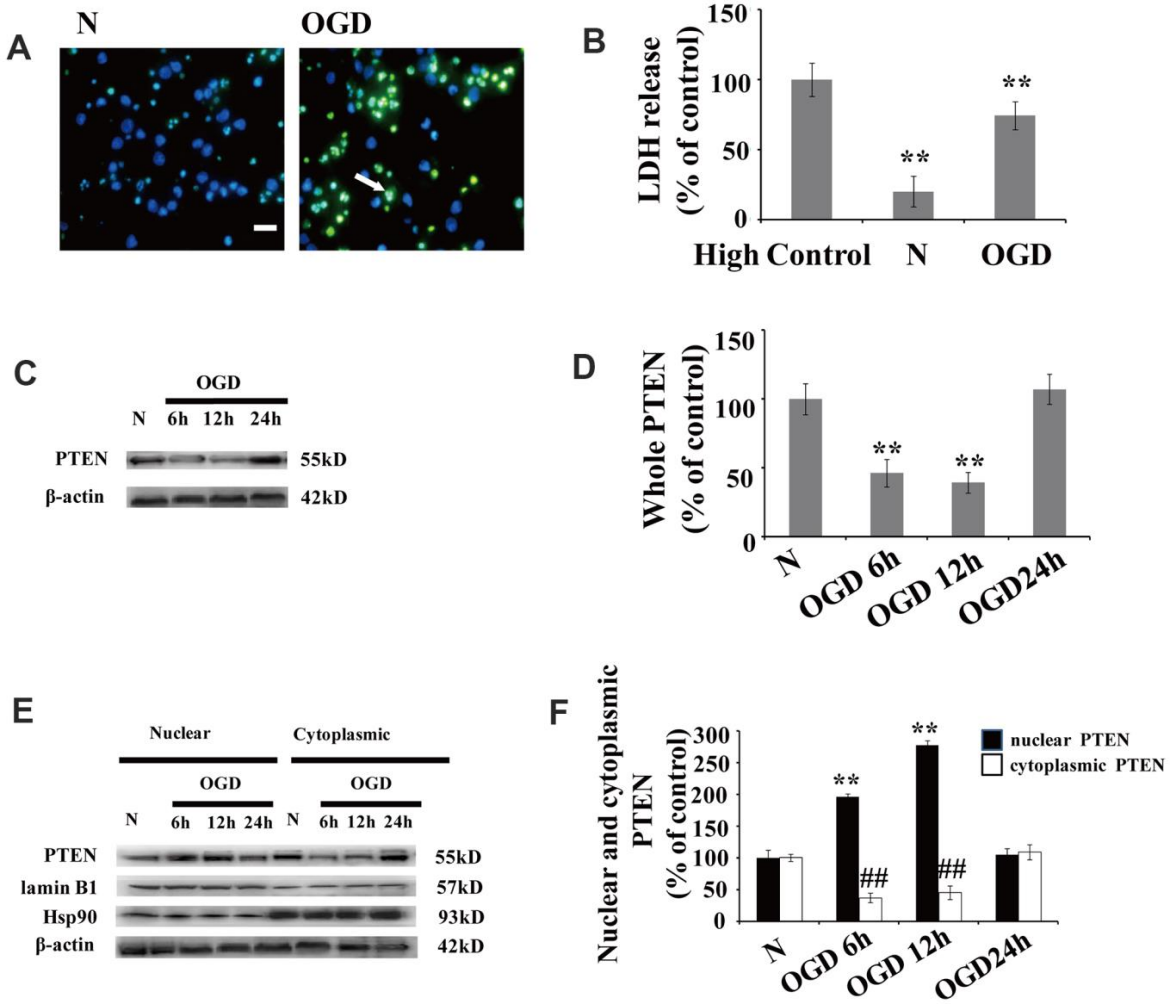
OGD increases PTEN nuclear translocation in cultured neurons (**A**) TUNEL-positive cells increased 12 h after OGD. The arrow indicates TUNEL-positive cells with green fluorescence. Scale bars = 50 μm. (**B**) Extracellular LDH levels were elevated at 12 h after OGD. (**C**) The PTEN whole cell protein levels decreased between 6 h and 12 h after OGD. (**D**) Quantification of the whole cell protein levels of PTEN. (**E**) Western blot analysis of the cytoplasmic and nuclear fractions showed that nuclear PTEN significantly increased 6 h after OGD, reaching a peak at 12 h, and returned to control levels within 24 h. Blots were re-probed using cytoplasmic and nuclear protein markers, Hsp90 and lamin B1, respectively. (**F**) Quantification of PTEN protein levels in cytoplasmic and nuclear fractions. Data were quantified by densitometry and normalized against healthy neurons. n = 5 for each column. **p < 0.01 vs. normal neurons. ## p < 0.01 vs. normal neurons. OGD: oxygen and glucose deprivation; N, normal neurons; TUNEL, terminal deoxynucleotidyl transferase-mediated dUTP-biotin nick-end labeling.

Significant neuronal damage was observed in cortical neuron cultures following OGD. The number of neurons with condensed/fragmented nuclei (Figure 1A) and levels of extracellular lactate dehydrogenase (LDH) (Figure 1B) increased after OGD.

After cytoplasm/nuclei fractionation, western blot was used to determine the effect of OGD on PTEN nuclear translocation. The protein blots were re-probed with corresponding cytoplasmic and nuclear protein markers, Hsp90 and lamin B1, respectively (Figure 1C). Cultured neurons showed a remarkable increase in nuclear PTEN after OGD. The protein level of nuclear PTEN was significantly increased at 6 h after OGD, reaching a peak at 12 h, and returned to control levels within 24 h (Figure 1C, 1D), which indicates that PTEN nuclear translocation is induced in response to OGD in a time-dependent manner. In contrast, cytoplasmic PTEN decreased between 6 h and 12 h post OGD, returning to control levels within 24 h (Figure 1C, 1D). A similar PTEN expression pattern was observed in whole cell extracts (Figure 1E, 1F).

### OGD inhibits the phosphorylation of ERK1/2 and NF-κB activation

**Figure 2:**
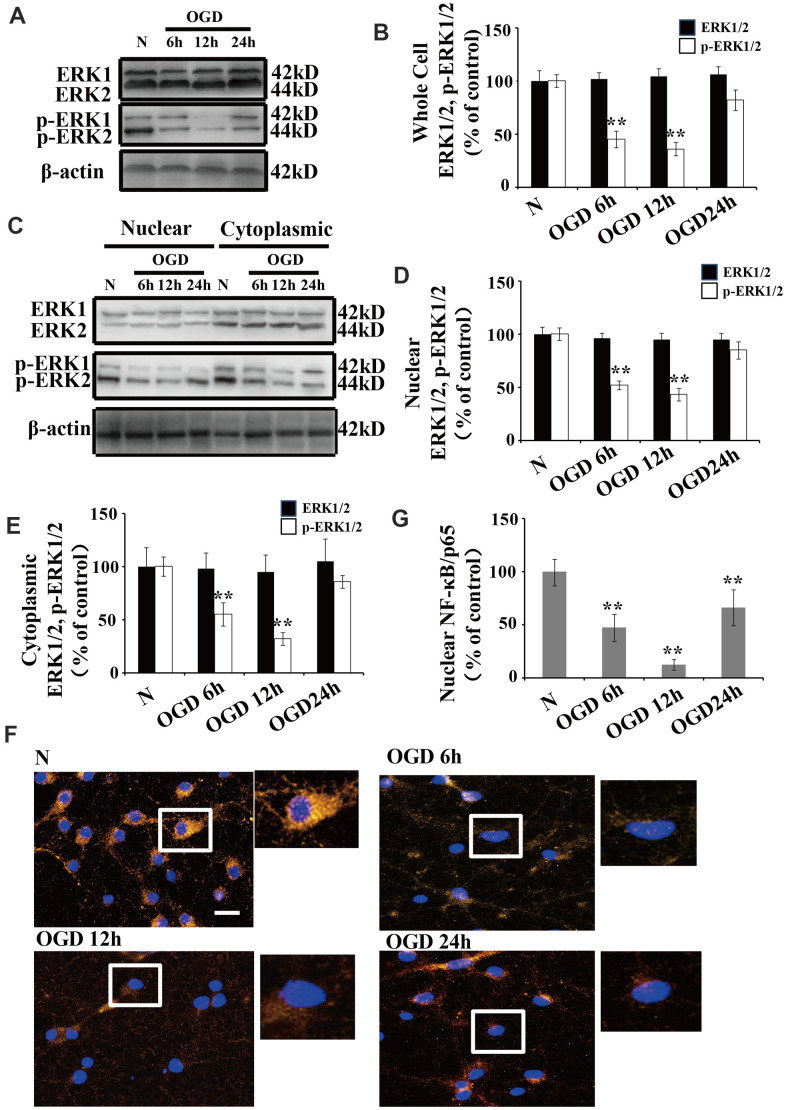
**OGD inhibits the phosphorylation of ERK1/2 and NF-κB activation.** (**A**) Whole cell protein levels of total ERK after OGD. The expression of p-ERK1/2 in whole cell extracts was significantly decreased 6 h after OGD, peaked at 12 h, and recovered at 24 h. (**B**) Quantification of ERK1/2 and p-ERK1/2 expression in whole cell extracts, normalized against normal neurons. (**C**) The expression of p-ERK1/2 in cytoplasmic and nuclear fractions. Western blot shows that nuclear p-ERK1/2 significantly decreased 6 h after OGD, peaked at 12 h, and recovered at 24 h. The changes in p-ERK1/2 expression in the cytoplasm was similar to that of the nucleus. (**D**) Quantification of nuclear ERK1/2 and p-ERK1/2 expression, normalized against normal neurons. (**E**) Quantification of cytoplasmic ERK1/2 and p-ERK1/2 expression, normalized against normal neurons. (**F**) Immunofluorescence staining of nuclear NF-κB. The nuclear translocation of NF-κB decreased after OGD, with minimal presence in the nucleus at 12 h post treatment. The arrow indicates nuclear-NF-κB/p65-positive cells with red fluorescence. (**G**) Qualification of fluorescence intensity of nuclear NF-κB staining, normalized against normal neurons. n = 5 in each column and **p < 0.01, vs. normal neurons. OGD: oxygen and glucose deprivation.

There were no significant changes in ERK1/2 expression following OGD when we compared the treated neurons to those cultured under normoxic conditions (Figure 2A, 2B). Similarly, there were no significant changes in ERK1/2 expression in the cytoplasmic and nuclear fractions following OGD (Figure 2C, 2D). However, the p-ERK1/2 level was significantly decreased at 6 and 12 h after OGD treatment in both the whole cell extracts (Figure 2A, 2B) and the nuclear-cytoplasmic fractions (Figure 2C–2E). The protein level of p-ERK1/2 in the nuclear fractions was significantly decreased at 6 h post OGD, peaked at 12 h, and then returned to baseline at 24 h (Figure 2C, 2D). The p-ERK1/2 expression patterns were similar in both the cytoplasmic and whole cell extracts (Figure 2A, 2C, 2E).

We used immunofluorescent staining to understand the changes in the subcellular distribution of NF-κB after OGD. NF-κB was primarily expressed in the cytoplasm of normal neurons, while a small amount of NF-κB was observed in the nucleus (Figure 2F). Time course experiments showed that the nuclear translocation of NF-κB significantly declined after OGD, with a minimal presence in the nucleus at 12 h (Figure 2F, 2G).

### The effects of PTEN nuclear translocation on NF-κB activity are mediated by the ERK signaling pathway

To determine whether nuclear PTEN inhibits ERK1/2 phosphorylation and NF-κB activation, neurons were transfected with PTEN_K13R_ mutant with a reduced ability to translocate into the nucleus. As PTEN nuclear translocation was markedly increased at 12 h after OGD, these analyses were carried out at 12 h post OGD.

**Figure 3:**
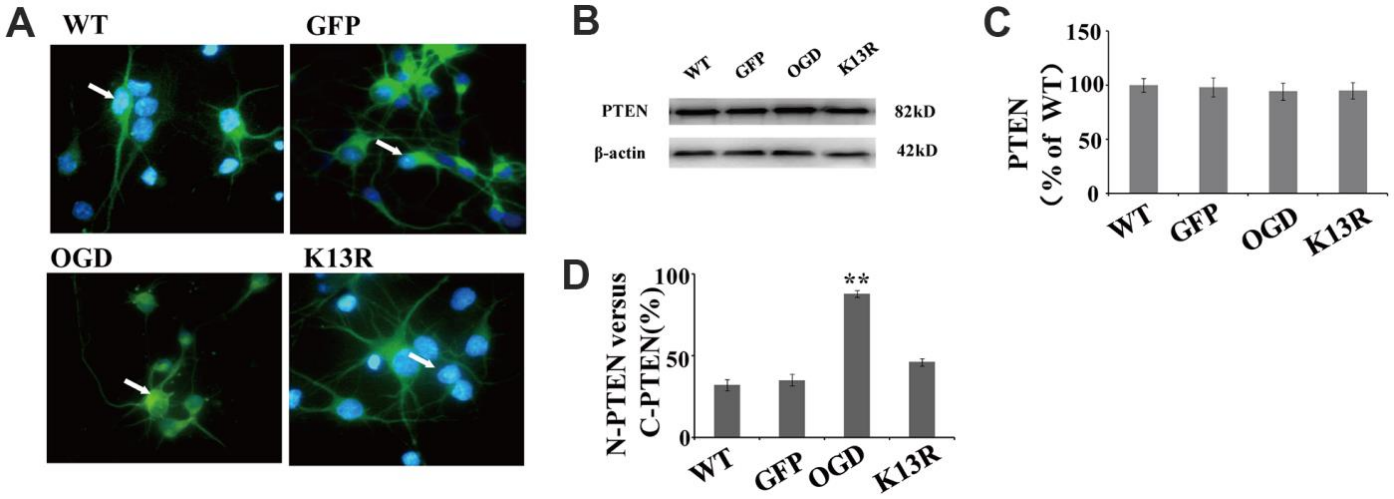
**OGD promotes PTEN nuclear translocation.** PTEN nuclear translocation is an essential step in the modulation of ERK1/2 and NF-κB activation. (**A**) Immunofluorescence staining of GFP in GFP-PTEN_K13R_ neurons. The GFP signal was predominantly cytoplasmic, and seldom observed in the nucleus. (**B**) The whole cell protein levels of PTEN in PTEN_K13R_ neurons. Western blot identified a normal sized PTEN protein in whole cell extracts. (**C**) Qualification of fluorescence intensity of PTEN, normalized against GFP-PTEN_WT_ neurons. (**D**) Qualification of fluorescence intensity of nuclear PTEN versus cytoplasmic PTEN, normalized against GFP-PTEN_WT_ neurons. n = 5 in each column and ** p < 0.01, vs. WT. GFP, green fluorescent protein; OGD: oxygen and glucose deprivation, WT: GFP-PTEN_WT_ neurons, K13R: GFP-PTEN_K13R_ neurons; P: PDTC.

When green fluorescent protein (GFP)-PTEN-_WT_ was transfected into neurons, the GFP signal was evenly distributed in both the nuclear and cytoplasmic compartments (Figure 3A). After OGD, this GFP signal was shown to be significantly enriched in the nucleus. Conversely, neurons transfected with the PTEN_K13R_ mutant plasmid (GFP-PTEN_K13R_) demonstrated a marked increase in cytoplasmic GFP signal, with relatively little PTEN_K13R_ observed in the nucleus. Western blot analysis showed that PTEN protein expression in PTEN_K13R_-transfected cells was comparable to that in the PTEN_WT_-transfected cells (Figure 3B), and this result was consistent with the fluorescence intensity data for GFP-PTEN (Figure 3C). After OGD, the ratio of GFP signal in the nucleus versus the cytoplasm was markedly increased and the ratio in the PTEN_K13R_ transferred cells were comparable to PTEN_WT_ cells (Figure 3D), which suggests that OGD promotes PTEN nuclear translocation which can be reversed by PTEN_K13R_.

**Figure 4:**
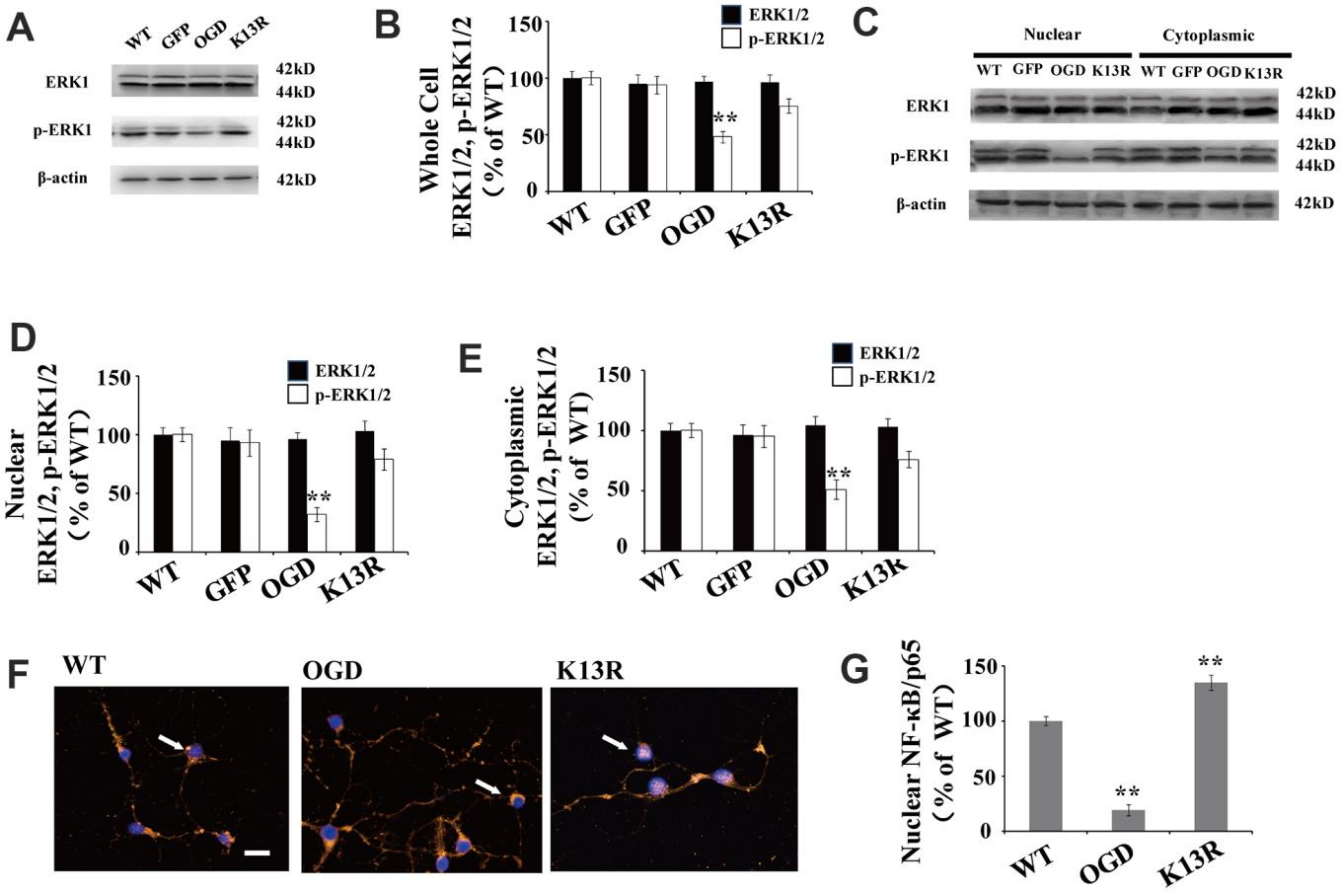
**PTEN nuclear translocation is an essential step in the modulation of ERK1/2.** (**A**) Whole cell protein levels for total ERK and p-ERK1/2. There were no significant changes in total ERK protein levels but p-ERK1/2 levels increased significantly in GFP-PTEN_K13R_ neurons after OGD. (**B**) Quantification of whole cell ERK1/2 and p- ERK1/2 expression, normalized against GFP-PTEN_WT_ neurons. (**C**) The expression level of p-ERK1/2 in cytoplasmic and nuclear fractions. Western blot showed that p-ERK1/2 expression increased in both the nuclear and cytoplasmic fractions in GFP-PTEN_K13R_ neurons after OGD. (**D**) Quantification of nuclear ERK1/2 and p-ERK1/2 expression, normalized against GFP-PTEN_WT_ neurons. (**E**) Quantification of cytoplasmic ERK1/2 and p-ERK1/2 expression, normalized against GFP-PTEN_WT_ neurons. (**F**) Immunofluorescence staining of nuclear NF-κB. The nuclear translocation of NF-κB increased in GFP-PTEN _K13R_ neurons after OGD. The arrow indicates nuclear-NF-κB-positive cells with red fluorescence. (**G**) Qualification of fluorescence intensity of nuclear NF-κB, normalized against GFP-PTEN_WT_ neurons. n = 5 for all columns and ** p < 0.01, vs. WT. OGD: oxygen and glucose deprivation, WT: GFP-PTEN_WT_ neurons; K13R: GFP-PTEN_K13R_ neurons.

There were no obvious changes in total ERK1/2 levels in GFP-PTEN_K13R_ neurons after OGD compared with those in GFP-PTEN_WT_ neurons (Figure 4A, 4B).

However, p-ERK1/2 expression in the GFP-PTEN_K13R_ neurons increased in both the whole cell extracts and nuclear-cytoplasmic fractions after OGD (Figure 4A–4E). Nuclear translocation of NF-κB was elevated in GFP-PTEN_K13R_ neurons (Figure 4F, 4G). Taken together, these findings indicate that PTEN nuclear translocation increases ERK1/2 and NF-κB activation following OGD in an *in vitro* model.

**Figure 5:**
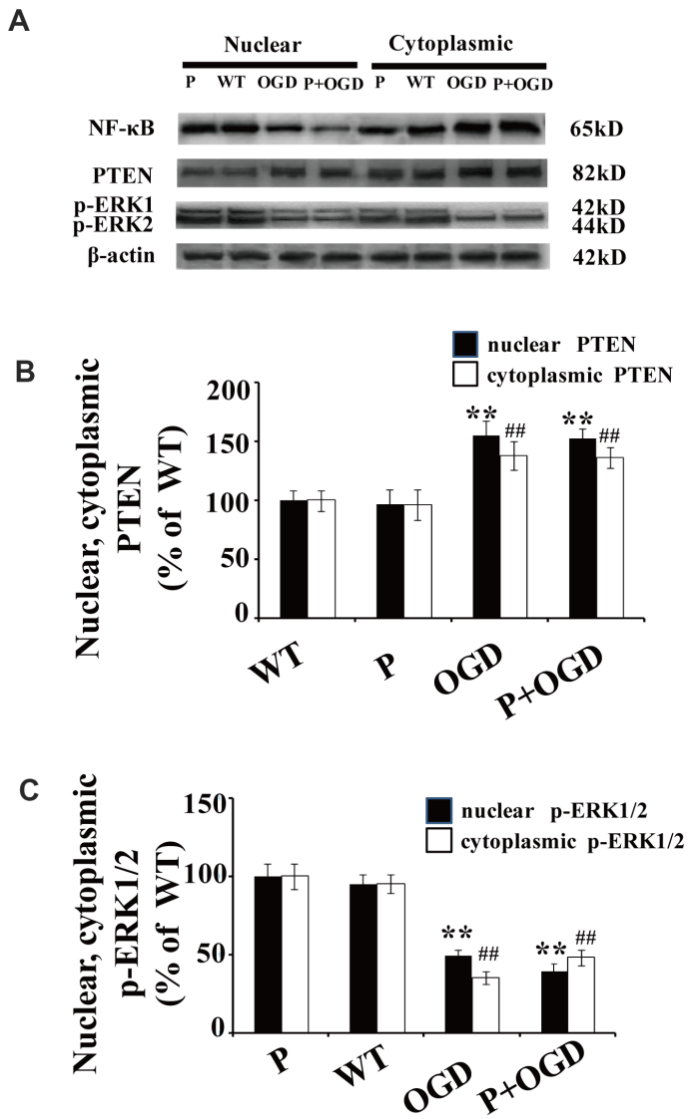
**PTEN nuclear translocation is an essential step in NF-κB activation.** (**A**) The expression of NF-κB and p-REK1/2 after PDTC treatment. Western blot of cytoplasmic and nuclear fractions shows that the nuclear translocation of NF-κB decreased in PDTC-treated neurons, while there was no obvious change in the nuclear translocation of PTEN or the expression of p-ERK1/2. (**B**) Quantification of nuclear and cytoplasmic PTEN expression, normalized against normal neurons. (**C**) Quantification of nuclear and cytoplasmic p-ERK1/2 expression, normalized against GFP-PTEN_WT_ neurons. n = 5 in each column and ** p < 0.01, vs. WT; ##p < 0.01, vs. WT. OGD: oxygen and glucose deprivation, WT: GFP-PTEN_WT_ neurons, K13R: GFP-PTEN_K13R_ neurons; P: PDTC.

Cultured cortical neurons were pretreated with 100 μM pyrrolidine dithiocarbonate (PDTC), a specific NF-κB inhibitor. The nuclear translocation of NF-κB decreased in PDTC-treated neurons (Figure 5A), while there were no significant changes in the nuclear translocation of PTEN and p-ERK1/2 in these cells (Figure 5A–5C).

### Inhibition of PTEN nuclear translocation reduces neuronal injury after OGD

**Figure 6:**
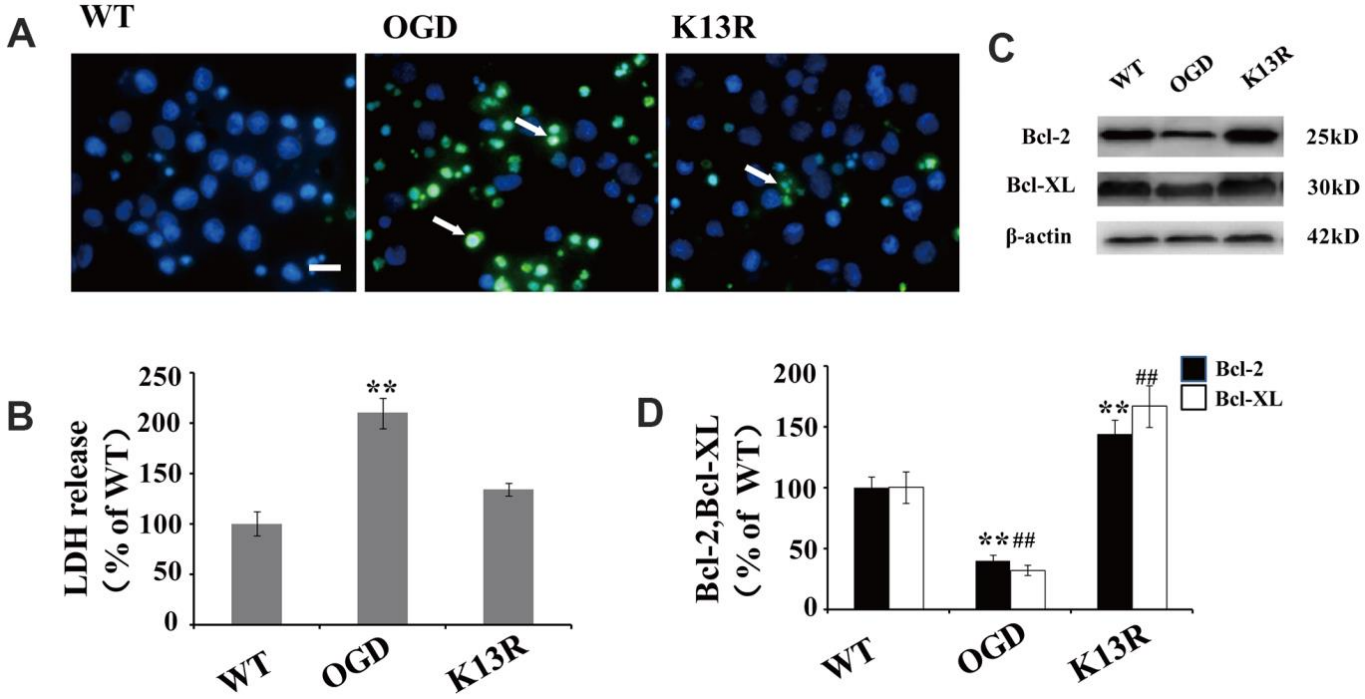
**Inhibition of PTEN nuclear translocation reduces neural injury after OGD.** (**A**) TUNEL-positive cells increased after OGD. This enhanced apoptosis was blocked in GFP-PTEN_K13R_ neurons. Scale bars = 50 μm. (**B**) Extracellular levels of LDH decreased in GFP-PTEN_K13R_ neurons after OGD. (**C**) Bcl-2 and Bcl-xL protein expression was evaluated by western blot which showed that their expression increased in GFP-PTEN_K13R_ neurons after OGD. (**D**) Quantification of Bcl-2 and Bcl-xL protein expression normalized against GFP-PTEN_WT_ neurons. n = 5 in each column and ** p < 0.01 vs. WT. ##p < 0.01, vs. WT. OGD: oxygen and glucose deprivation, GFP: green fluorescent protein, TUNEL: terminal deoxynucleotidyl transferase-mediated dUTP-biotin nick-end labeling WT: GFP-PTEN_WT_ neurons, K13R: GFP-PTEN_K13R_ neurons; P: PDTC.

The potent effects of PTEN_K13R_ on PTEN nuclear translocation following OGD supports a causal role for PTEN in HI-induced neuronal injuries. The number of terminal deoxynucleotidyl transferase-mediated dUTP-biotin nick-end labeling (TUNEL)-positive cells significantly increased following OGD. This enhanced apoptosis was blocked in GFP-PTEN_K13R_ neurons (Figure 6A). Moreover, results from the LDH assay also support the hypothesis that inhibition of PTEN nuclear translocation exerts some neuroprotective effect during HI injury (Figure 6B).

As NF-κB plays a key regulatory role in the expression of various anti-apoptotic proteins, including Bcl-2 and Bcl-xL, the association between OGD-induced PTEN nuclear translocation and anti-apoptotic protein expression was investigated. Western blot analysis showed that the expression of Bcl-2 and Bcl-xL declined following OGD exposed cells (Figure 6C, 6D). However, an inverse effect was on the expression of these proteins was observed in PTEN_K13R_ neurons, suggesting that the inhibition of PTEN nuclear translocation after OGD could upregulate anti-apoptotic genes (Figure 6C, 6D).

## DISCUSSION

PTEN was first identified as a tumor suppressor gene and has been implicated in neuronal damage following excitotoxic excitotoxic/ischemic injury [24, 25]. Diao et al. showed that m6A-mediated activation of PTEN plays an important role in protecting neurons against neuronal hypoxia/re-oxygenation-induced pyroptosis [26]. In addition, PTEN-dependent autophagy in cerebral ischemia/reperfusion injuries has been linked to the neuroprotective activity of MTMR14 [27]. Shabanzadeh et al. suggests that human PTEN peptide treatment could induce functional improvements following mid-cerebral artery occlusion or retinal ischemia triggered by ophthalmic artery ligation [28]. Nuclear localization of PTEN is a dynamic process related to cell cycle progression and cellular differentiation, which can be triggered by cellular injuries such as ischemia [29]. However, the role of PTEN in the development of brain injuries after HI and the involved mechanisms are not fully understood. In this study, an *in vitro* OGD model was constructed using cultured neurons to mimic HI. We attempted to explore the possible mechanism underlying the activities of PTEN during brain damage following HI.

Our data shows that PTEN localizes to both the nuclear and cytoplasmic compartments of cortical neurons and that the increase in PTEN nuclear translocation is an essential step during the signaling cascades contributing to OGD-induced neuronal injuries. Similar findings have been reported in another recent study, which suggests that PTEN nuclear translocation plays an essential role in ischemia-induced neuronal injuries [11]. In our study, nuclear PTEN translocation increases significantly following OGD and coincides with a significant increase in the number of apoptotic cells in these cultures. These observations were shown to be reversed when cells were transfected with a GFP-PTEN_K13R_ mutant. These data indicated that the nuclear translocation of PTEN plays a causative role in HI-induced neuronal injuries.

Our data also shows that the nuclear translocation of PTEN plays a vital role in neuronal injury via the down-regulation of ERK1/2 and NF-κB activation. Previous evidence suggests that PTEN is an intrinsic factor in neurodegenerative diseases, such as Alzheimer's and Parkinson's diseases [22]. PTEN has been hypothesized as an important regulator of apoptosis and axon regeneration in HI-induced brain injury [30, 31]. The nucleocytoplasmic distribution of PTEN has been found to be responsible for its tumor suppressive function [8]. It has been reported that nuclear PTEN is associated with neuronal soma size and seizure susceptibility [9]. Here, we show that expression of PTEN in the whole cell and cytoplasmic fractions of injured neurons decreases between 6 h and 24 h post OGD, while the nuclear PTEN level increases during this time. These results suggest that the nuclear translocation of PTEN occurs after exposure to OGD. In addition, our data shows that increases in nuclear PTEN concentrations was time-dependent with a maximum concentration at 12 h post OGD. A previous study reported that the activity of cytoplasmic PTEN significantly increased at 3 h after OGD. Taken together, these data suggest that PTEN might be involved in delayed signaling following OGD.

Substantial evidence suggests that PTEN encodes a bipartite nuclear localization signal (NLS)-like sequence that mediates its nuclear import [32]. Several recent cancer studies have suggested that mono-ubiquitination of lysine residue K13 plays a critical role in mediating PTEN nuclear accumulation [33, 34].

Therefore, we investigated the roles of nuclear PTEN using a K13R PTEN mutant, which retains its normal lipid phosphatase activity but lacks the ability to translocate to the nucleus. Experiments using a myocardial ischemia/reperfusion model demonstrated that ERK signaling, and an increase in ERK phosphorylation, plays a protective role in preventing myocardial ischemia/reperfusion injury [35]. The ERK1/2 isoforms are most studied in neurological research. Thus, we targeted the ERK1/2 isoforms in this study. Our data shows a decreased level of ERK1/2 phosphorylation in both the cytoplasm and nucleus following OGD, which was consistent with the changes in nuclear PTEN expression. These findings suggest that decreasing p-ERK after OGD may be associated with increasing PTEN nuclear translocation.

NF-κB is a transcriptional regulator, known primarily for its role in inflammation, neuronal survival, apoptosis, and neurite growth [36–38]. Under normal environmental conditions, NF-κB dimers remain in the cytosol in an inactive state; however adverse environmental conditions, such as accumulation of β-amyloid (Aβ)/reactive oxygen species (ROS)/cytokines, induce the activation and nuclear translocation of the NF-κB dimers [39]. These activated NF-κB dimers are critical to the expression of anti-apoptotic proteins, such as Bcl-2 and Bcl-xL [40, 41]. It has been reported that the inhibition of ERK suppresses Aβ-induced NF-κB transactivation, leading to the inhibition of Aβ-induced neurotoxicity [42]. Here, we found that the nuclear translocation of NF-κB decreases following OGD, reaching the lowest levels at 12 h post injury; this finding is in agreement with the changes in the nuclear expression of both PTEN and ERK1/2. Collectively, these findings suggest that decreased NF-κB activity following OGD may be the result of increased PTEN nuclear translocation. In addition, when PTEN_K13R_ was transfected into the nucleus, the expression of nuclear p-ERK1/2 and NF-κB both increased following OGD. However, using PTDC to inhibit NF-κB activation did not change the nuclear translocation of PTEN and p-ERK1/2, suggesting that PTEN nuclear translocation modulates the activation of ERK1/2 and NF-κB following OGD.

Neurodegeneration can be caused by increased PTEN nuclear translocation-induced neuronal death [43]. Inhibited PTEN nuclear translocation may contribute to the pathogenesis of various cancers by promoting cell growth [44]. Here we characterized the underlying mechanisms of PTEN nuclear localization in response to OGD-induced HI and found that PTEN_K13R_ transfected neurons exhibited decreased neuronal vulnerability to ischemic insult. A decrease in extracellular levels of LDH was also shown to coincide with an increased expression of the anti-apoptotic proteins, Bcl-2 and Bcl-xL when PTEN nuclear translocation was inhibited. These results suggest that PTEN nuclear translocation inhibits ERK1/2 and NF-κB activation following OGD and that this inhibition correlates with a sequential decrease in the expression of the anti-apoptotic proteins regulated by NF-κB.

Despite the comprehensive nature of these findings, there were some limitations to our current study. First, the nuclear translocation of PTEN was only investigated at the cellular level, further *in vivo* experiments are needed to confirm these results *in vivo* to obtain a more reliable result. Second, fluorescence staining was not enough to investigate PTEN nuclear translocation. Nuclear export signals and nuclear localization signals will be investigated in the future. In addition, study had demonstrated that PTEN inhibition could enhance angiogenesis in OGD-exposure human umbilical vein endothelial cells by activating AKT signals cascade [45]. Wang et al. also showed PTEN inhibition was implicated in enhancing proliferation and angiogenesis of human brain microvascular endothelial cells [46]. Angiogenesis is a crucial pathophysiological response to cerebral ischemia. Whether angiogenesis involves the role of PTEN nuclear translocation to neuronal injury, it should be further investigated in the future.

In conclusion, our study provides insight into the molecular mechanisms through which PTEN nuclear translocation contributes to neuronal injury following HI. We found that PTEN nuclear translocation is an essential step in the activation of ERK1/2 and NF-κB in neurons subjected to OGD and that inhibition of PTEN nuclear translocation can provide protection against ischemic brain damage. PTEN_K13R_ mutants transfected into neurons effectively inhibited PTEN nuclear translocation, without affecting the cytoplasmic functions of PTEN. Taken together, these results suggest that the inhibition of PTEN nuclear translocation could be a potent novel strategy for protecting neurons following HI *in vivo*.

## MATERIALS AND METHODS

### Primary cortical neuron isolation and culture

Newborn Sprague-Dawley rats were obtained from the Experimental Animal Center of North Sichuan Medical College within 24 h of birth. Primary cortical neurons were then isolated from these newborn rats within another 24 h. The cerebral cortex tissues were extracted, and cells were dissociated in trypsin solution for 10 min at 37° C. These dissociated cells were seeded into six-well plates pre-coated with poly-D-lysine (Sigma), and the medium was replaced by neurobasal medium (NB; Gibco) supplemented with 2% B27 (Gibco) and 500 μM glutamine (Gibco). The primary neurons were maintained at 37° C in a 5% CO_2_ atmosphere. Our study was approved by the Animal Ethics Committee of the North Sichuan Medical College and all the animal procedures were performed in accordance with the animal protection guidelines.

### Establishment of the OGD model

The OGD model was established as previously described [47]. On day 7 of cell culture, the primary neurons were collected and washed with phosphate-buffered saline (PBS) twice. These cells were then maintained in Dulbecco’s Modified Eagle Medium without glucose and exposed to hypoxia (95% N_2_, 5% CO_2_) at 37° C in an air-tight chamber for 3 h. After 3 h of OGD, the cell culture medium was replaced by NB medium, and cells were returned to the normoxic incubator at 5% CO_2_ and 37° C.

### Plasmids and transfection

The pcDNA3.1 plasmid was prepared by Jinsite Biotechnology. *Xho*I and *BamHI* restriction sites were added to the 5’ and 3’ ends of the products, respectively. The sequence of PTEN was amplified using the forward primer 5’-CCGCTCGAGCAGTCGCTGCAACCATCCA-3’ and the reverse primer 5’-CGCGGATCCGACTTTTGTAATTTGTGTATGC-3’. The PTEN sequence was cloned into pcDNA3.1 and sub-cloned into pEGFP-C2 (Clontech, #6083-1) to produce GFP-PTEN. The GFP-PTEN_K13R_ plasmid was constructed using site-directed mutagenesis of GFP-PTEN_WT_, which introduced a lysine 13 residue (K13R), using the QuikChange Kit (Stratagene, #200518-5). The WT ubiquitin plasmid contains 8 tandem HA-ubiquitin repeats under the control of a CMV promotor. When evaluating PTEN protein expression, GFP-PTEN_K13R_ plasmid was transferred into neurons using a Baculovirus system and GFP-PTEN_WT_ was used as a control.

Neural transfection was completed using DIV5 and the X-tremeGENE HP DNA Transfection Reagent (Roche) according to the manufacturer’s instructions. Following transfection, neurons were incubated in 5% CO_2_ at 37° C for 48 h. The inhibition of PTEN nuclear translocation was confirmed by immunofluorescence (IF) staining.

### Nuclei fractionation

The nuclear and cytoplasmic fractions of cultured cortical neurons were separated using the Nuclear/Cytosol Fractionation Kit (Biovision, Catalog #: K266) and the cytoplasmic and nuclear fractions were confirmed by detecting heat shock protein 90 (Hsp90) and lamin B1, respectively.

### Western blot

Neural proteins were extracted using a protein lysis buffer (Thermo, USA) and then, 100 μg of these proteins were separated on an 8% sodium dodecyl sulfate-polyacrylamide gel using SDS-PAGE. These proteins were then transferred to a nitrocellulose (NC) membrane (Bio-Rad) which was blocked in 5% skim milk for 1 h at room temperature. These membranes were then incubated overnight at 4° C with the appropriate primary antibody: rabbit PTEN polyclonal antibody (1:1000; Cell Signaling), rabbit phosphorylated-PTEN (p-PTEN) (Ser380) polyclonal antibody (1:1000; Cell Signaling), rabbit ERK1/2 polyclonal antibody (1:1000, Abcam), rabbit phospho-p44/42 ERK1/2 (Thr202/Tyr204) monoclonal antibody (1:2000; Cell Signaling), mouse p-IκB-α monoclonal antibody (Ser 32) (1:500; Santa Cruz Biotechnology), rabbit NF-κB (p65) polyclonal antibody (1:1000; Beyotime), rabbit B cell lymphoma-2 (Bcl-2) polyclonal antibody (1:1000, Abcam), and rabbit B cell lymphoma extra-large (Bcl-xL) polyclonal antibody (1:500, Abcam). These membranes were then incubated with the secondary antibody, horseradish peroxidase-conjugated immunoglobulin G (1:3000; Santa Cruz Biotechnology), at room temperature for 1 h. Protein expression was then evaluated using an enhanced chemiluminescence system (Millipore).

### PDTC treatment

PDTC ammonium salt (Biovision) was dissolved in double distilled (dd) H_2_O and stored at -20° C. Before OGD treatment, cultured neurons were pretreated with PDTC (100 μM) for 24 h and then kept under constant PDTC exposure for the 24 h following OGD model construction.

### LDH assay

In order to evaluate neuronal injury after OGD, the extracellular LDH level in positive control (High Control), normal (N) and OGD groups (OGD) was quantified using a toxicology assay kit (Sigma-Aldrich) as per the manufacturer’s instructions. The results were analyzed using spectrophotometric absorbance at 490 nm.

### TUNEL assay

The (TUNEL assay was performed to detect apoptotic neurons in both the normal (N) and OGD groups (OGD) using the In-Situ Cell Death Detection Kit from Roche (Mannheim, Germany). Images were observed using a fluorescence microscope (Leica DM IRB Olympus BX61). The apoptotic cells were counted in 10 random fields at a magnification of 400 x. The apoptotic index (AI) was calculated using the following equation: AI = (number of apoptotic cells/total number of cells) x 100%.

### Immunofluorescence analysis

Primary cell cultures were fixed in 4% paraformaldehyde at 4° C for 20 min and treated with 0.1% Triton X-100 for 15 min. After cells were treated with 10% serum, they were incubated in the appropriate primary antibodies, including rabbit PTEN antibody (1:100; Cell Signaling) and rabbit NF-κB (p65) antibody (1:100; Beyotime) overnight at 4° C. After washing in PBS cells were incubated with secondary antibodies conjugated to fluorescein or Texas Red (Vector Labs, Burlingame, CA, USA) at room temperature for 30 min and then counterstained with 4,6-diamidino-2-phenylindole dihydrochloride (DAPI) for 5 min. These cells were then observed under a fluorescence microscope (Nikon, TiE).

### Statistical analysis

SPSS software v17.0 (IBM, USA) was used for statistical analysis. All quantitative data are displayed as the mean ± standard deviation (SD). Comparisons among multiple groups were performed using one-way ANOVA followed by a Bonferroni post hoc test. Significance was set at a p-value of <0.05.
